# Analyzing the role of CagV, a VirB8 homolog of the type IV secretion system of *Helicobacter pylori*

**DOI:** 10.1002/2211-5463.12225

**Published:** 2017-05-24

**Authors:** Navin Kumar, Mohd Shariq, Amarjeet Kumar, Rajesh Kumari, Naidu Subbarao, Rakesh K. Tyagi, Gauranga Mukhopadhyay

**Affiliations:** ^1^ Special Centre for Molecular Medicine Jawaharlal Nehru University New Delhi India; ^2^ School of Computational and Integrative Sciences Jawaharlal Nehru University New Delhi India; ^3^ Present address: School of Biotechnology Gautam Buddha University Yamuna Expressway Greater Noida Gautam Budh Nagar Uttar Pradesh India; ^4^ Present address: School of Life Sciences Jawaharlal Nehru University New Delhi India

**Keywords:** CagA, *cag*‐PAI, Cag‐T4SS, CagV, VirB8

## Abstract

The type IV secretion system of *Helicobacter pylori* (Cag‐T4SS) is composed of ~ 27 components including a VirB8 homolog, CagV. We have characterized CagV and reported that it is an inner membrane protein and, like VirB8, forms a homodimer. Its stability is not dependent on the other Cag components and the absence of *cagV* affects the stability of only CagI, a protein involved in pilus formation. CagV is not required for the stability and localization of outer membrane subcomplex proteins, but interacts with them through CagX. It also interacts with the inner membrane‐associated components, CagF and CagZ, and is required for the surface localization of CagA. The results of this study might help in deciphering the mechanistic contributions of CagV in the Cag‐T4SS biogenesis and function.

**Abbreviations**
DSP: dithiobis succinimidyl propionate
IFM: immunofluorescence microscopy
IP: immunoprecipitation
MBP: maltose‐binding protein
RMSD: root mean square deviation
T4SS: type IV secretion system
TEM: transmission electron microscopy
Y2H: yeast two hybrid

Type I strains of *Helicobacter pylori* (*H. pylori*) harboring type IV secretion system (Cag‐T4SS) are responsible for the more severe forms of gastric diseases 1, 2, 3, 4. Cag‐T4SS is composed of ~ 27 components and encoded by a ~ 37‐kb genomic region called *cag*‐Pathogenicity Island (*cag*‐PAI) 5. *H. pylori* uses this specialized secretion system to translocate the substrate CagA into the host epithelial cells to modulate the host signaling pathways and to induce proinflammatory response 6, 7, 8, 9.

The most studied prototypical secretion system, the VirB/D4 T4SS of *Agrobacterium tumefaciens* (*A. tumefaciens*) is composed of 11 VirB proteins (VirB1‐VirB11) and a coupling protein VirD4 10, 11. Several Cag proteins have sequence similarity to the VirB/D4 components and are considered as homologs, like CagY as VirB10; CagX as VirB9; CagT as VirB7; CagV as VirB8 etc. 12, 13. Additionally, Cag‐T4SS encodes a number of unique components like Cagδ, CagZ, CagU, and CagM etc. 12, 13, 14, 15. VirB proteins that are involved in the assembly of the secretion system could be subdivided into three groups 16. Cag‐T4SS is also presumed to have similar type of organization of the Cag components as shown in a number of hypothetical models 12, 13, 16, 17. However, a recent report has shown that the architecture of the core Cag‐T4SS complex is significantly different from that of other published T4SS core complexes 18. Very little is, however, known about the architecture of the system and the molecular mechanism of substrate translocation across the bacterial membranes.

In the present study, we have characterized the VirB8 homolog CagV which shares weak sequence homology with its counterpart in other known T4SS. However, on the basis of topological analysis in *E. coli*, it is predicted to be a VirB8 homolog, an essential component of the VirB/D4 secretion system in *A. tumefaciens* 19, 20. In *H. pylori*, mutational analysis revealed that CagV is essential for CagA translocation and IL‐8 induction in the host cells 5, 21.

In *A. tumefaciens*, VirB8 is a bitopic membrane protein and is required for the stability of several other VirB proteins underlining its importance in the biogenesis of the secretion complex 22, 23. Hence, VirB8 is suggested to be an assembly factor that is connected to the outer membrane core complex via VirB9 and VirB10 24, 25. It is also connected to the cytoplasmic core complex via VirB4 and to the periplasmic protein VirB1 for breaking down peptidoglycan layer to facilitate the T4SS assembly across the bacterial cell envelope 19, 26, 27, 28. In order to understand the molecular basis of native CagV function in the Cag‐T4SS assembly and substrate transportation to the bacterial surface, we have investigated its subcellular localization, its interactions with *cag*‐PAI components and compared the findings with its counterpart VirB8 of *A. tumefaciens*.

## Materials and methods

### Bacterial strains and growth conditions

All the bacterial strains used in this study are listed in Table 1. Wild‐type *H. pylori* and mutant strains were grown and maintained as described earlier 14. *E. coli* cultures were also grown as described earlier 14.

**Table 1:** *H. pylori* and *E. coli* strains used in the present study

Strains	Descriptions	References
WT 26695	*Helicobacter pylori* wild‐type strain	Tomb *et al*. 1997 30
*∆cagZ*	Deletion mutant for *cagZ*	Kumar *et al*. 2013 29
*∆cagV*	Deletion mutant for *cagV*	This study
*∆cagT*	Deletion mutant for *cagT*	Fischer *et al*. 2001 5
∆*cagX*	Deletion mutant for *cagX*	Kumar *et al*. 2013 29
∆*cagA*	Deletion mutant for *cagA*	Fischer *et al*. 2001 5
*∆cagM*	Deletion mutant for *cagM*	Kumar *et al*. 2013 29
*∆cagY*	Deletion mutant for *cagY*	Kumar *et al*. 2013 29
*∆cag*δ	Deletion mutant for *cag*δ	Shariq *et al*. 2015 14
Δ*cagV/cagV*	Complementation strain of *cagV*	This study
*E. coli BL21(DE3) pLys*	Expression host for pET28a	Promega Corporation, Madison, WI, USA
*E. coli DH5*α	General cloning host	NEB, Ipswich, MA, USA

### Cloning, construction of mutant strains, and expression of genes

All primers used in this study are mentioned in Table 2. pET‐28a was used to express CagV (His tag) and pMal‐c2X was used to express CagV and CagF with maltose‐binding protein (MBP) tag. Proteins were expressed in *E. coli* strain BL‐21 (DE3) at 25 °C in LB medium with 0.1 mm IPTG (isopropyl‐β‐D‐1‐thiogalactopyranoside) to induce the expression of proteins in the culture medium. All *cag* null mutant strains were constructed in *H. pylori* 26695, as described previously except *cagV* 5, 14, 29. To generate the nonpolar *cagV* null mutant strain, mutator DNA was synthesized by overlapping PCR using a *cagV* upstream fragment containing the first three codons of *cagV* and the upstream region of *cat* cassette (using primers DV1 and DV3), the downstream region of *cagV* containing the termination codon, and the downstream region of *cat* cassette (using primers DV2 and DV4) and amplified *cat* cassette (using primers Fcat and Rcat). Finally a linear mutator PCR product was synthesized using primers DV1 and DV2 and phusion polymerase. Next, the resulting linear PCR product was introduced into *H. pylori* 26695 by natural transformation following a published protocol and selected on chloramphenicol BHI (Brain Heart Infusion) agar plate 14. Complementation of the wild‐type *cagV* was performed as described earlier 14. Briefly, the *cagV* (under *cagA* promoter)‐containing plasmid was transformed into Δ*cagV* and expressed in the presence of appropriate antibiotics and tested for CagV expression.

**Table 2:** Oligonucleotides used in the present study

F*cagV*Bam	5′‐CGGGATCCATGTTAGGGAAAAAAAACG ‐3′
R*cagV*Xho	5′‐CCGCTCGAGCTATTTATTTAATGCCTTATTTTTTG‐3′
R*cagV*Sal	5′‐ATGCGTCGACCTATTTATTTAATGCCTTATTTTTTG‐3′
F*cagF*Bam	5′‐CCGGATCCATGAAACAAAGTTTGCGC‐ 3′
R*cagF*PstI	5′‐AAAACTGCAGATCGTTACTGTTGTTTTGATT‐ 3′
F*cagV*cBam	5′‐CCGGATCCATGTTAGGGAAAAAAAACGAA‐3′
R*cagV*cKpnI	5′‐CAGGGGTACCTTATTTATTTAATGCCTTATTTTTTGA‐3′
DV1	5′‐AACTCCCTTTAGGATATAATGAGT‐3′
DV2	5′‐TGTCCTCAACACCGCCTT‐3′
DV3	5′‐CTTCCTTAGCTCCTGAAAATCTCGCCCTAACATGCGACAGCT‐3′
DV4	5′‐TGGCAGGGCGGGGCGTAAGAACATGTTTAATATTAAAAGGAATTT‐3′
F*cat*	5′‐GTTTTTGGATCCATCCGAGATTTTCAGGAGCTA‐3′
R*cat*	5′‐AAAAATTACGCCCCCGCCCTTTAGG‐3′

### Antibody generation, SDS/PAGE, and western blotting

Polyclonal antibodies against Cag proteins such as Cagδ, CagH, CagL, CagE, CagY, CagZ, CagX, CagT, CagM, CagI, and CagF were raised by immunization of rabbit and mice in the laboratory as described previously 14, 29. Briefly, to generate polyclonal antibody against CagV, a full‐length *cagV* was cloned in pET‐28a vector and overexpressed in *E. coli* strain BL‐21 (DE3). CagV protein band was excised from SDS/PAGE, used for immunization, specificity and titer of rabbit anti‐CagV (α‐CagVr) and mice anti‐CagV antibody (α‐CagVm) were tested by western blotting. Anti‐CagA, anti‐OMP (Outer Membrane Protein), and anti‐HSP (Heat Shock Protein) antibodies were from Santa Cruz, CA, USA (Cat. No. Sc‐25766, Sc‐69935 and Sc‐57779, respectively). Anti‐MBP antibody was from NEB, USA (Cat. No. E8032L). SDS/PAGE and western blotting were performed as described previously 14, 29.

### Ethics statement

The present study was approved by the Institutional Animal Ethics Committee‐of Jawaharlal Nehru University (Code no: 23/2007 and 22/2012). The animals (Balb/c mice female or New Zealand white rabbit female) were maintained at Central Animal Facility of the Jawaharlal Nehru University as approved by the Institutional Animal Ethics Committee. After experimental procedures were finished, the animals were maintained until their natural death and every effort was made to minimize their suffering.

### Cell fractionation

Cellular fractionation of the wild‐type *H. pylori* cells was performed as described earlier 14. Briefly, bacterial cell extract was centrifuged at 148 000 ***g*** in SW‐55 rotor (Beckman coulter ultracentrifuge, Irving, TX, USA) at 4 °C for 1 h, the supernatant was recovered as cytoplasmic/periplasmic fraction (C/P) and the resulting pellet was recovered as total membrane (TM). Volume of C/P was measured and TM was resuspended in the same volume of PBS. Equal volume of each sample was mixed with 2X‐SDS sample buffer, separated in SDS/PAGE, and subjected to western blotting using an appropriate antibody.

### Immunofluorescence microscopy (IFM)

*Helicobacter pylori* cells were immunostained and viewed as described earlier 29. Briefly, fixed cells were incubated with specific polyclonal antibody of appropriate dilutions (α‐CagV‐1 : 500, α‐CagZ‐1 : 500, α‐CagT‐1 : 1000, α‐CagX‐1 : 1000, and α‐CagA‐1 : 1000) and respective preimmune serum (negative control).

### Immunoprecipitation (IP)

*Helicobacter pylori* cell extract was used for coimmunoprecipitation study as described previously 14. Briefly, bacterial cells (~ 100 μL cell pellet volume) were lysed in 1 mL of lysis buffer (1× PBS pH 7.4, 2 mm EDTA, 2 mm DTT, 1% TritonX‐100, 0.2% sodium deoxycholate, 1 mg·mL^−1^ lysozyme, and 6 μL of 100X‐protease inhibitor cocktail), sonicated, centrifuged, and the supernatant was collected and precleared with preimmune serum and protein‐A agarose beads (G. Biosciences, St. Louis, MO, USA, Cat. No. 440P). Next, DMP (Dimethyl Pimelimidate; Cat. No. 21667) cross‐linked anti‐CagV antibody with protein‐A agarose beads (20 μL) was added to the precleared supernatant and incubated at 4 °C O/N on rocker. Following brief centrifugation, beads were washed three times with lysis buffer without lysozyme, bound proteins were released by boiling in SDS sample buffer and subjected to western blotting.

### Purification of MBP tagged proteins and pull‐down assay

For purification of recombinant proteins (MBP‐tagged CagV and CagF), *E. coli* cell pellets containing expressed proteins were resuspended in 50 mm Tris‐HCl, pH 8.0, 150 mm NaCl, 2 mm EDTA, 1 mm PMSF, 1% TritonX‐100, 0.2% sodium deoxycholate, 2 mm DTT, and 1 mg·mL^−1^ lysozyme, lysed by sonication, and centrifuged at 15 700 ***g*** for 30 min. The supernatant was diluted with an equal volume of the above buffer without lysozyme (binding buffer), prewashed amylose–agarose beads were added and incubated at 4 °C for 2 h. Beads were washed with binding buffer five times and proteins were eluted with elution buffer (20 mm maltose in binding buffer), dialyzed against 50 mm Tris‐HCl pH 7.4, 1 mm EDTA, and 5% glycerol, protein concentration was measured using CBXtm kit (Genotech), and analyzed in SDS/PAGE. For pull‐down assay, purified MBP‐tagged CagV and CagF or MBP alone bound to amylose–agarose beads (10 μg each; NEB, Cat. No. E8021S) were mixed with *H. pylori* cell extract (1 mg·mL^−1^) prepared in binding buffer (50 mm Tris‐HCl, 150 mm NaCl, pH 7.4, 1% TritonX‐100, 0.2% sodium deoxycholate, 2 mm DTT, 2 mm EDTA, and 1 mm PMSF, 6 μL of 100X‐protease inhibitor cocktail), O/N. Next, protein‐bound beads were collected by centrifugation, washed three times with binding buffer, proteins were eluted by boiling in 2X‐SDS sample buffer, separated in SDS/PAGE, and western blotted.

### Transmission electron microscopy (TEM)

Transmission electron microscopy was performed on an ultrathin section of *H. pylori* cells as described earlier 14. Primary antibodies were used at a dilution of 1 : 100. Cells were negatively stained with 4% phospho‐tungstate uranyl acetate (pH 4.0) and examined under a JEM‐2100F (JEOL) transmission electron microscope.

### Cross‐linking of *H. pylori* cells

Plate‐grown fresh *H. pylori* cells were resuspended in 500 μL of lysis buffer (150 mm NaCl, 2 mm EDTA, and 1 mm PMSF in PBS). Cells were ruptured by sonication. Thereafter, 0.1% DDM (*n*‐Dodecyl β‐D‐maltoside; Cat. No. D5172) and 0.2% sarcosine were added to the extract, incubated for 30 min on ice, 5 mm DSP [dithiobis (succinimidyl propionate), G. Biosciences‐BC07] were added and further incubated on ice for 25 min. The cross‐linking reaction was stopped by adding Tris‐HCl (pH 7.4) to a final concentration of 125 mm, incubated for 15 min on ice, centrifuged at 15 700 ***g*** at 4 °C for 15 min, and the supernatant was collected.

### Comparative sequence analysis

Multiple sequence alignment (MSA) of *H. pylori* CagV [(*H. pylori*‐Cag10) (Uniport ID: Q6VRI3)], *A. tumefaciens*‐VriB8 (Uniprot ID: P17798), and *Brucella suis* (*B. suis*) VirB8 (Uniprot ID: Q7CEG3) was performed using Clustal Omega and the alignment was visualized and analyzed using jalview (v.2.0) to identify conserved regions in the periplasmic domain of CagV 31, 32.

### Homology modeling and molecular dynamics simulation of CagV monomer and dimer

The sequence of CagV corresponding to the crystallized periplasmic domains of *A. tumefaciens*‐VirB8 (92‐237 aa) and *B. suis*‐VirB8 (97‐234 aa) was identified. A multiple template‐based model of the identified region was generated using Modeller (v9.14) 33, 34. The highest resolution crystal structures of *A. tumefaciens*‐VirB8 (PDB ID: 2CC3) and *B. suis*‐VirB8 (PDB ID: 4AKZ) were taken as templates 35, 36. Structure with lowest discrete optimized potential energy was used to obtain a more stable state for molecular dynamics (MD) simulation 37. MD was performed using gromacs (v5.1) and gromos96 54a7 force field 37, 38, 39, 40. Cubical box filled with the simple point charged water models was used for solvation, under periodic boundary conditions with padding distance of 1.5 nm, keeping protein at center. Few water molecules were replaced by required number of counter ions (Na^+^/Cl^−^) needed to neutralize the system at pH 7.0. A maximum of 10 000 steps of steepest descent was performed to minimize the energy of the system. Solvent and ionic molecules were equilibrated around the protein under NVT and NPT conditions simultaneously (for 200 ps each), to maintain 300 K temperature and 1 bar pressure. For this step a positional restraint was applied on the protein. Finally, a 70‐ns long unrestrained production MD was run with time step of 2 fs at 300 K and 1 bar pressure. Energy and the co‐ordinates were saved every 10 ps (at every 5000 step) for further analysis. The final step co‐ordinates of CagV obtained after simulation was superimposed on both the chains of *A. tumefaciens*‐VirB8 (PDB ID: 2CC3, Chain A and B), one by one and transformed co‐ordinates were saved and combined to generate the dimer of CagV. Chain Identifier A and B were assigned to the chains. Initial interchain clashes were removed manually by slightly shifting the chains apart and introducing a slight rotational shift in UCSF chimera 41. Co‐ordinates of the dimer were then subjected to a 50‐ns MD simulation following similar procedure applied to the monomer. The conserved interchain contacts at the dimer interface throughout the simulation were calculated using mdcons package (v2.0). It considers a contact by a cut‐off distance of 5 Å between heavy atoms of two residues of different chains at the interface 42.

The buried solvent accessible surface area throughout simulation at the dimer interface was calculated by subtracting the total solvent accessible surface area (SASA) of the CagV dimer from the sum of total SASA of each individual monomeric units of the dimer. The buried hydrophilic and hydrophobic surface areas were also calculated using the same approach. gromacs tool gmx sasa was used to calculate SASA and the residues having charge between −0.2 and 0.2 were treated as hydrophobic, while others as hydrophilic. The plot of interaction across the interface of final CagV dimer co‐ordinate for *A. tumefaciens*‐VirB8 and *B. suis*‐VirB8 was generated using dimplot (v.1.4.5) 43. The binding free energies of complexes were calculated using dcomplex package (http://sparks-lab.org/czhang/complex.html) 44. Other trajectory analysis and visualization was carried out using inbuilt gromacs tools, ucsf chimera (v‐1.10.2, (http://www.cgl.ucsf.edu/chimera, and vmd v‐1.9.2, http://www.ks.uiuc.edu/Research/vmd/) 41, 45. Graphs were generated using grace software package (v‐5.1.23, http://plasma-gate.weizmann.ac.il/Grace/).

## Results

### Cellular localization of CagV

**Figure 1:**
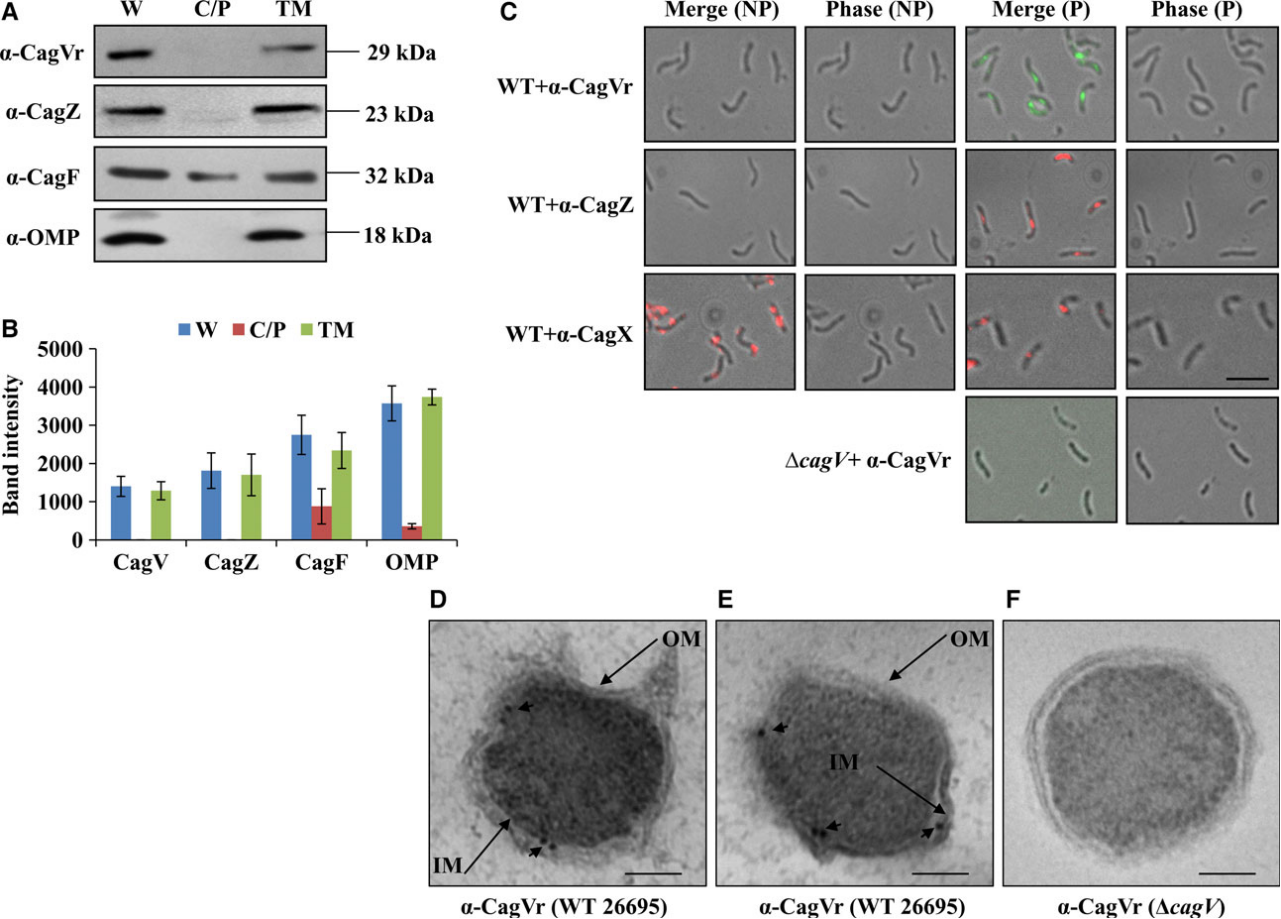
Subcellular localization of CagV in *H. pylori*. (A) Western blots showing subcellular fractionation of wild‐type *H. pylori*. W, C/P, and TM indicate whole cell lysate, soluble (cytoplasmic/periplasmic), and total membrane fractions, respectively. Inner membrane protein CagZ was used as a control for membrane protein, whereas CagF was used as a control for both the soluble and membrane proteins. OMP was used as a loading control. Vr indicates anti‐CagV rabbit antibody. (B) Intensity of individual protein band is measured from three independent experiments by densitometry using imagej software and plotted. Data are represented as mean ± SD. (C) IFM showing CagV localization in the wild‐type *H. pylori* cells under permeabilized (P) and nonpermeabilized (NP) conditions. Δ*cagV* mutant strain was used as a negative control. CagZ and CagX were used as negative and positive controls. Primary antibodies are indicated. Alexa fluor 488‐ (green color) and Alexa fluor 594 (red color)‐conjugated secondary antibodies were used for signal detection. Magnification bar indicates 5 μm. Out of 650 cells having fluorescent foci tested, 150 foci were detected at the poles, 400 foci were detected at the middle of the bacterium, and the remaining 100 foci were detected near the poles. (D and E) Representative TEM images showing the localization of CagV in the wild‐type *H. pylori*. Ultrathin sections of wild‐type cells were immunostained using anti‐CagV antibody and gold‐labeled secondary antibody (particle size 15 nm). (F) TEM image showing absence of CagV in Δ*cagV* strain (negative control). Arrow heads indicate location of gold‐labeled secondary antibody. Arrows indicate position of inner and outer membranes (IN and OM). Magnification bars indicate 100 nm. Multiple ultrathin sections (16 sections) were scanned. Number of gold‐labeled particles attached to the inner membrane was determined and found to be 5 ± 2. Data are represented as mean ± SD.

Following *in silico* analysis of the transmembrane domain of CagV and expression of the corresponding gene in *E. coli,* Buhrdorf *et al*. predicted that *cagV* might be a functional *virB8* analog 20. Later, it was accepted as a VirB8 homolog. However, not much is known about the protein. In order to understand the location of native CagV, we performed cell fractionation of the wild‐type *H. pylori* 26695 into soluble cytoplasmic/periplasmic (C/P) and total membrane fractions (TM). The fractions were separated in SDS/PAGE, western blotted using appropriate antibodies and quantitated (Figs 1A,B and S1). As shown in Fig. 1A, like inner membrane‐associated CagZ, CagV was also found exclusively in the membrane fraction 46, 47, 48. Subsequently, IFM analysis of wild‐type *H. pylori* under permeabilized (P) and nonpermeabilized (NP) conditions supported its localization inside the cell (Fig. 1C). These results, thus, suggest that CagV is not a bacterial surface exposed protein rather; it resides inside the bacterium and is associated with membrane. To visualize exact localization of the protein we performed TEM and detected CagV‐specific signals at the inner membrane suggesting its inner membrane association (Fig. 1D,E).

### Stability of CagI is partially affected in the absence of CagV

**Figure 2:**
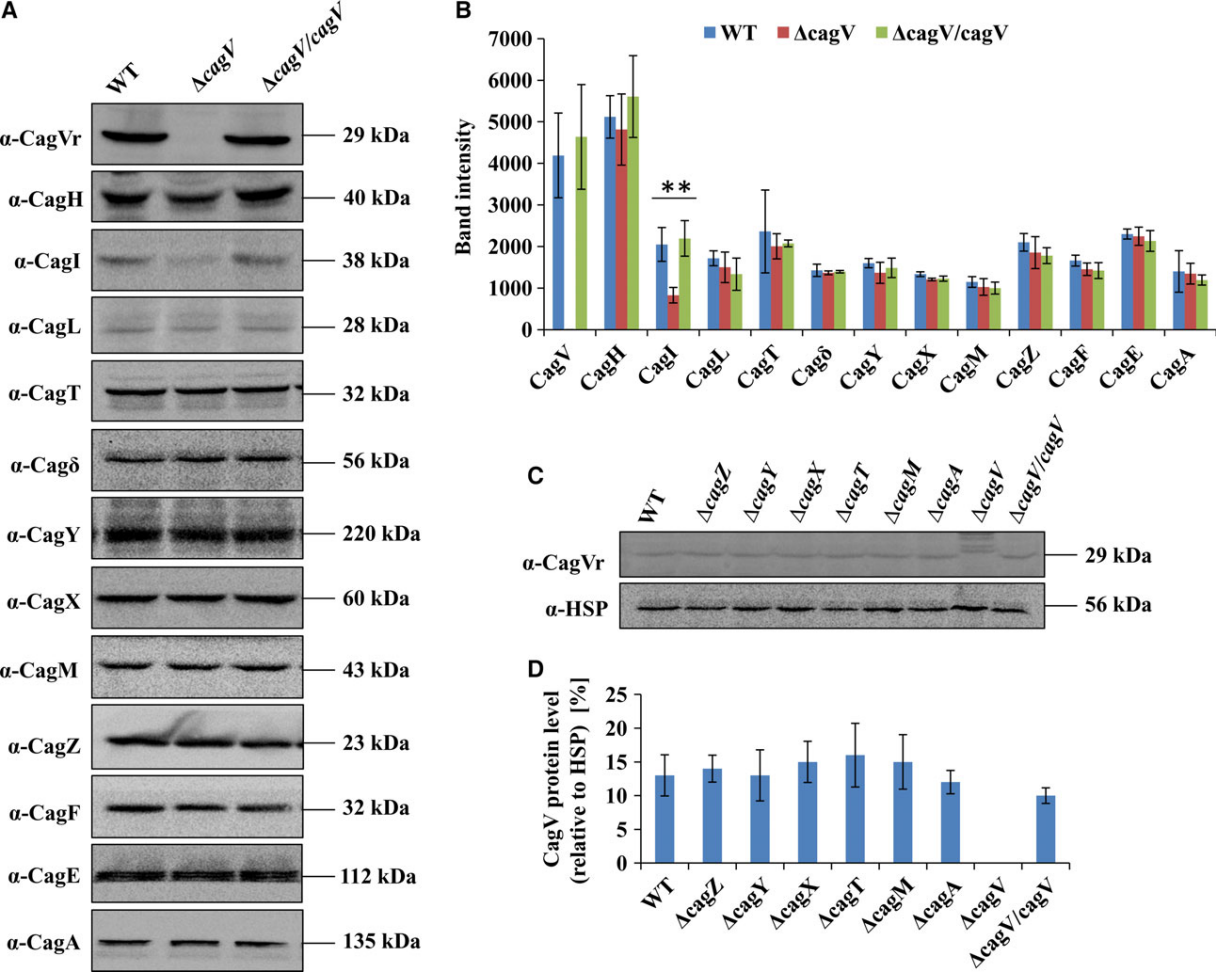
Western blots showing stability of T4SS proteins in Δ*cagV* mutant strain. (A) Western blots showing the stability of several essential Cag proteins including components required for pilus formation in wild‐type *H. pylori*, Δ*cagV*, and *cagV* complemented Δ*cagV/cagV* strains. Equal amounts of proteins were loaded in SDS/PAGE, separated and western blotted. Antibodies used are marked. Vr indicates anti‐CagV rabbit antibody. (B) Intensity of individual protein band is measured from three independent experiments by densitometry using imagej software and plotted. The statistical analyses were performed using Student's *t*‐test. The differences were considered statistically significant when *P* is < 0.05. **Signifies *P* ≤ 0.01. Data are represented as mean ± SD. (C) Stability of CagV in the absence of indicated *cag* mutants. HSP was used as a loading control. Antibodies used are marked. (D) Intensity of individual protein band is measured from three independent experiments by densitometry using imagej software and plotted with respect to HSP (%). Data are represented as mean ± SD.

To understand the role of CagV in the stability of Cag components, we tested the levels of individual Cag proteins in the absence of *cagV* in Δ*cagV* by western blot analysis. As shown in Fig. 2A,B, all the tested structural components of Cag‐T4SS such as CagT, Cagδ, CagY, CagX, CagM, and CagZ along with CagE, chaperone CagF, and substrate CagA were found stable. However, the stability (level) of CagI, the protein involved in the Cag‐T4SS pilus formation was found to be partially affected (Fig. 2A,B). Stability of no other known protein involved in pilus formation, like CagH and CagL (VirB5 homolog) were affected. Complementation of the *cagV* function in Δ*cagV/cagV* supported the result.

In the case of the prototypical T4SS of *A. tumefaciens*, stabilities of several VirB proteins are reported to be affected in the absence of VirB8. For example, the stability of VirB3 and VirB6 are strongly reduced in its absence, whereas modest effect is observed in the cases of VirB1, VirB4, VirB5, VirB7, and VirB11 22. Among these, VirB3 and VirB5 are involved in the T pilus biogenesis 49. However, when the stability (level) of CagV was tested in the absence of other Cag components, no apparent change in CagV stability was observed (Fig. 2C,D). It is worth mentioning that in *A. tumefaciens*, however, VirB8 stability is dependent on VirB4, an energy providing protein 50.

### CagV‐independent stability and surface localization of CagT and CagX

**Figure 3:**
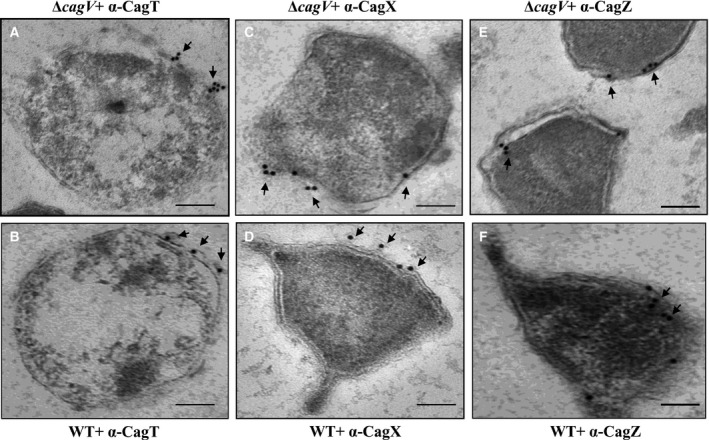
TEM showing surface localization of CagT and CagX in wild‐type *H. pylori* and Δ*cagV* strains. Ultrathin sections of wild‐type *H. pylori* and Δ*cagV* cells were prepared and immunostained as described in the Materials and methods. (A) Δ*cagV* stained with anti‐CagT antibody. (B) Wild‐type *H. pylori* stained with anti‐CagT antibody. (C) Δ*cagV* stained with anti‐CagX antibody. (D) Wild‐type *H. pylori* stained with anti‐CagX antibody. (E) Δ*cagV* stained with anti‐CagZ antibody. (F) Wild‐type *H. pylori* stained with anti‐CagZ antibody. Gold‐labeled secondary antibody (particle size 15 nm) was used to visualize the respective antigens. CagZ was used as an inner membrane protein marker. Magnification bars indicate 100 nm. Arrowheads indicate location of gold‐labeled particles. Multiple ultrathin sections of indicated cells (15 sections for each panel) were scanned. Number of gold‐labeled particles on the surface/attached to the outer membrane was counted and found to be 5 ± 2 and 4 ± 2 for CagT in Δ*cagV* and wild‐type cells, respectively. For CagX, the values were 4 ± 2 in both Δ*cagV* and wild‐type cells, respectively. Statistical analyses were performed using Student's *t*‐test and differences found to be statistically insignificant for both CagT and CagX in the strains tested. The differences were considered statistically insignificant when *P* ≥ 0.05. Data are represented as mean ± SD.

**Figure 4:**
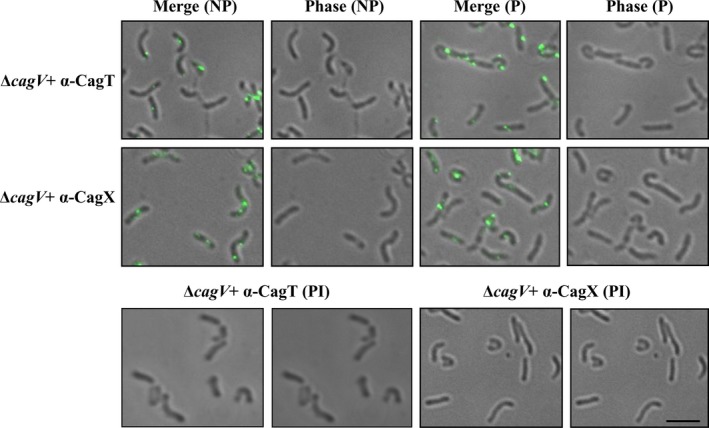
IFM showing localization of CagT and CagX in Δ*cagV* strain. Bacterial cells were harvested from BHI‐agar plate, fixed, and permeabilized with 0.2% TritonX‐100 as described in Materials and methods. NP and P stand for nonpermeabilized and permeabilized cells, respectively. Primary antibodies used in IFM are indicated. Preimmune serum (PI) was used as a negative control. Alexa fluor 488 (green color)‐conjugated secondary antibody was used for detection of the antigens. Magnification bar indicates 5 μm. Out of 340 Δ*cagV* cells (from three different experiments) tested, CagT specific fluorescence signals were detected in 250 cells under NP and out of 390, 310 cells showed fluorescence under P conditions. Similarly, out of 490 Δ*cagV* cells tested, CagX‐specific signals were detected in 355 cells under NP and out of 620, 500 cells showed fluorescence under P conditions.

In the prototypical VirB/D4 T4SS and others, VirB8 protein is reported to be an essential assembly factor of the secretion systems 19, 50, 51, 52. We, thus, asked the question whether CagV like VirB8 affects the stability and localization of the key outer membrane subcomplex proteins of the Cag‐T4SS, such as CagT and CagX. As mentioned above, CagV does not affect the stability of the Cag proteins except CagI. We, therefore, tested the localization of these proteins in the wild‐type *H. pylori* and isogenic Δ*cag*V by IFM and TEM. As shown in Figs 3 and 4, like in the wild‐type *H. pylori* cells, CagT‐ and CagX‐specific signals were also observed in the isogenic Δ*cag*V. Taken together, these results thus suggest that not only the stability but also the native location of the proteins are independent of CagV. This is in contrary to the VirB/D4 T4SS, where stability, polar localization, and assembly of the components are dependent on VirB8 22.

### CagV‐dependent surface localization of CagA

**Figure 5:**
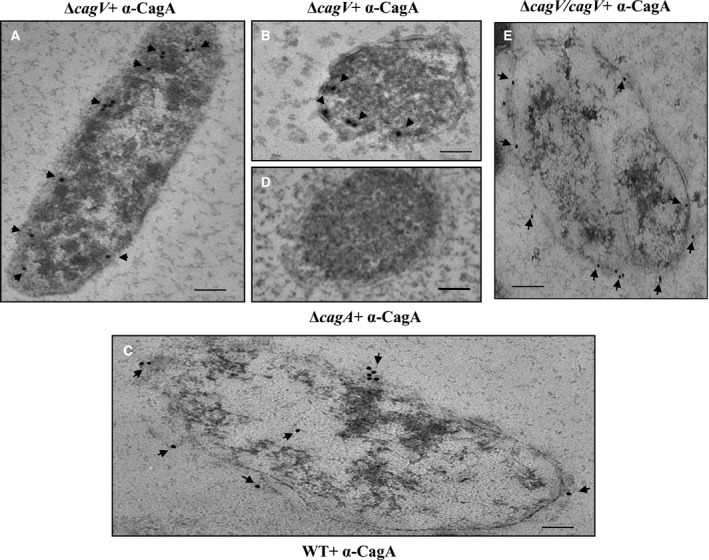
CagV‐dependent surface localization of CagA. TEM images showing localization of CagA in wild‐type *H. pylori,* Δ*cagV*, and Δ*cagV/cagV*. (A and B) Ultrathin sections of Δ*cagV,* (C) Wild‐type *H. pylori,* (D) Δ*cagA* and (E) Δ*cagV/cagV* cells were immunostained with anti‐CagA antibody. Δ*cagA* was used as a negative control. Gold‐labeled secondary antibody (particle size 15 nm) was used to visualize the respective antigens. Magnification bars indicate 100 nm. Arrowheads indicate location of gold‐labeled secondary antibody. Multiple ultrathin sections (20 in wild‐type, 28 in Δ*cagV* and 20 in Δ*cagV*/*cagV*) were scanned. Number of gold‐labeled particles attached to the inner membrane or on the surface/attached to the outer membrane were counted and found to be 5 ± 3, 6 ± 2, and 6 ± 3, respectively. Data are represented as mean ± SD.

Recent studies have shown that the Cag‐T4SS substrate CagA is a cell surface‐exposed protein 14, 53, 54. To investigate the role of CagV in the translocation of CagA to the bacterial surface we determined the localization of CagA in the absence of CagV in ∆*cag*V. We performed IFM on the wild‐type *H. pylori* and ∆*cag*V cells under permeabilized (P) and nonpermeabilized (NP) conditions. As shown in Fig. S2, unlike in wild‐type *H. pylori*, the CagA‐specific signal was not detected in the Δ*cag*V under NP condition. However, when IFM was performed under permeabilized condition, the CagA‐specific signal was detected in the Δ*cag*V cells. These results thus suggest that CagV may be required for the bacterial surface localization of CagA. To further validate the above data, we complemented the *cagV* function in the Δ*cag*V, performed IFM, and observed that CagA again reappeared on the bacterial cell surface like in wild‐type *H. pylori*. We further performed TEM on the wild‐type *H. pylori,* isogenic Δ*cag*V and Δ*cag*V/*cagV* strains. In agreement with the IFM results, CagA‐specific signals were detected in the cytoplasmic face of the inner membrane along the perimeter in the Δ*cag*V (Fig. 5A,B). However, under a similar condition CagA‐specific signals were detected associated with the outer membrane and on the bacterial cell surface in the wild‐type and *cagV* complemented Δ*cag*V/*cagV* strains (Fig. 5C,E). CagA‐specific signals were, however, absent in Δ*cag*A strain (used as a negative control; Fig. 5D). Taken together, these results suggest that CagV is essential for CagA translocation across the bacterial membranes.

### Comparative sequence analysis and homology modeling of CagV monomer

Comparative sequence analysis of VirB8 homologs has shown that despite having low sequence identity, several of the amino acid residues are conserved across the family members and participate in the interaction either with itself or with other VirB proteins or DNA substrate 35, 55. We, therefore, tested the formation of CagV dimer in SDS/PAGE followed by western blotting. However, we failed to observe any dimer. This result thus prompted us to perform *in silico* homology modeling of CagV using the crystal structure data of *A. tumefaciens*‐VirB8 and *B. suis*‐VirB8.

**Figure 6:**
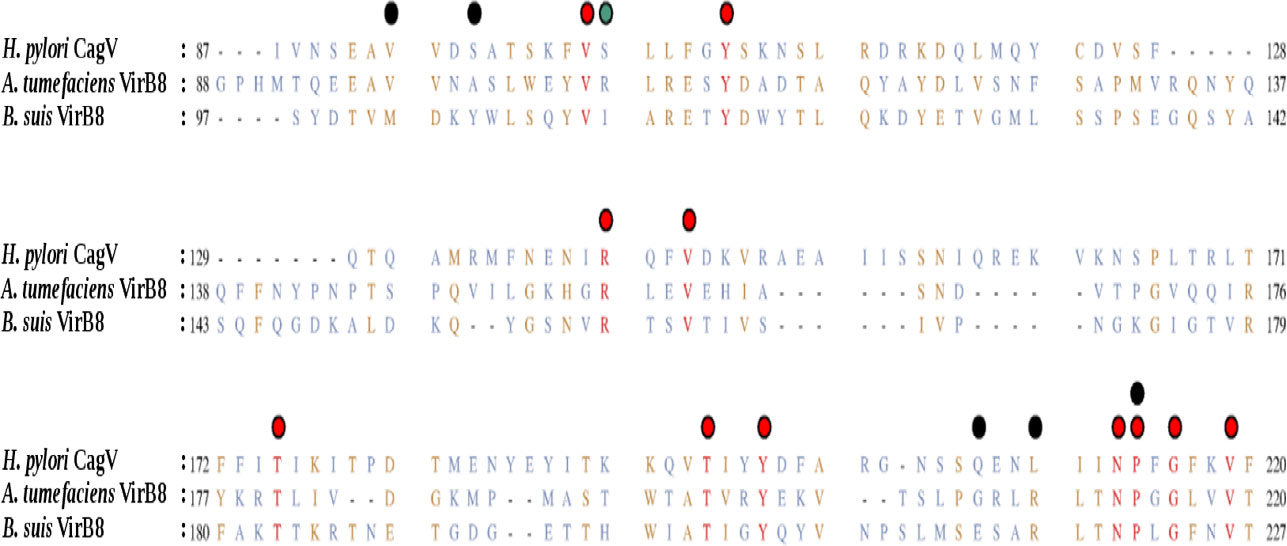
Sequence analysis of the CagV periplasmic domain. Multiple sequence alignment of CagV (*H. pylori*‐Cag10) with its two homologs: *B. suis*‐VirB8 and *A. tumefaciens*‐VirB8. Sequence coloring is as per degree of conservation. Strictly conserved residues (conserved in all three sequences): red; moderately conserved residues (conserved in any two sequences): golden yellow; conserved in none: grayish blue. Red dots represent conserved residues in all three homologs and black dots represents reported essential residues for self dimerization in either of the VirB8 homologs.

Studies on the *B. suis*‐VirB8 periplasmic domain identified amino acids required for protein–protein interactions and homodimerization 56. Similarly, in *A. tumefaciens*‐VirB8 periplasmic domain, V97 and A100 were reported essential for homodimerization 22. Comparative sequence and structural analysis showed that CagV shares 17% and 19% identity with *A. tumefaciens*‐VirB8 and *B. suis*‐VirB8, respectively (Fig. 6). We observed a strictly conserved region 210‐LxxNPxGxxV‐219 which is equivalent to 210‐RxxNPxGxxV‐219 of *A. tumefaciens*‐VirB8 and 217‐RxxNPxGxxV‐227 of *B. suis*‐VirB8. Other conserved residues are shown on the Fig. 6.

**Figure 7:**
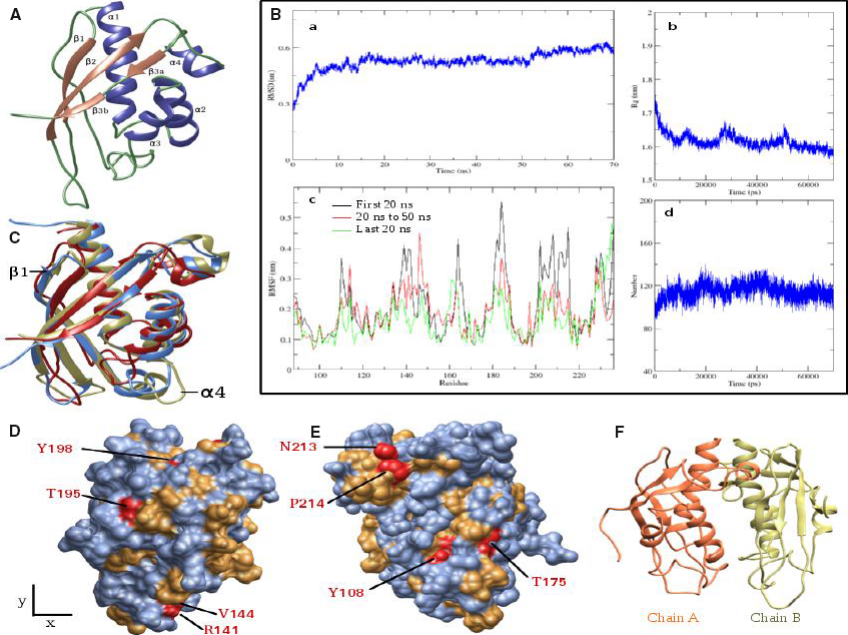
Structural analysis of CagV periplasmic domain. (A) Structure of CagV periplasmic domain (87‐228 aa). α‐helices are shown in indigo, β‐sheets are shown in light brown and coils are shown in light green. (B) MD simulations of CagV monomer. (a) RMSD with reference to modeled structure. (b) Radius of gyration. (c) Residue wise RMSF for the first 20 ns, 20–50 ns, and last 20 ns. (d) Number of intrachain hydrogen bonds. (C) Structural alignment of CagV monomer with VirB8 of *A. tumefaciens* and *B. suis*. CagV: Brick red, *A. tumefaciens*‐VirB8 (PDB ID: 2CC3, Chain A): Light blue and *B. suis*‐VirB8 (PDB ID: 4AKZ, chain A): Kakhi. α4 helix and β1 strand of *A. tumefaciens*‐VirB8 are labeled. (D) Surface representation of CagV. Surface color is as per degree of conservation. Residue coloring is as shown in Fig. 6, orientation as in A. (E) CagV surface view obtained by rotating D 180° around *y* axis and 20° around *x* axis anti‐clock wise. Most conserved residues on the surface are labeled in D and E. (F) Cartoon representation of the modeled CagV homodimer.

We generated a homology model of CagV periplasmic domain (87‐236 aa) by using *A. tumefaciens*‐VirB8 and *B. sui*s‐VirB8 as templates (Fig. 7A). It shared 18% average identity with periplasmic domains of the templates. The modeled structure was further subjected to a 70‐ns‐long molecular dynamics simulation to obtain its stable conformation. Convergence was observed in root mean square deviation (RMSD) value, radius of gyration, and number of intrachain hydrogen bonds, 15 ns onwards (Fig. 7B). Looking at the residue wise RMSF curve, it is clear that fluctuations in amino acid positions during the last 20 ns of the run has decreased as compared to the first 20 ns and 20–50 ns (Fig. 7B). These observations point that the structure of CagV has attained a conformation with high compactness and less fluctuations which is comparatively more stable than the modeled structure. The co‐ordinates of the final stable structure were saved for further analysis. It has an RMSD of 1.147 Å and 1.118 Å with *A. tumefaciens*‐VirB8 and *B. suis*‐VirB8 and consists of four α‐helices (α1, α2, α3, and α4) juxtaposed by antiparallel β‐sheets (β1, β2 and β3) (Fig. 7A,C). Unlike *A. tumefaciens*‐VirB8 and *B. suis*‐VirB8, β‐sheets are relatively smaller in size. One β strand and one α‐helix corresponding to β1 and α4 of *A. tumefaciens*‐VirB8 have found to be transformed into coils (Fig. 7C). Eight of the conserved amino acids are exposed on the surface and are accessible to solvent (Fig. 7D,E).

### MD simulation of CagV homodimer, comparison of dimer interfaces, and *in vitro* formation of CagV dimer

**Figure 8:**
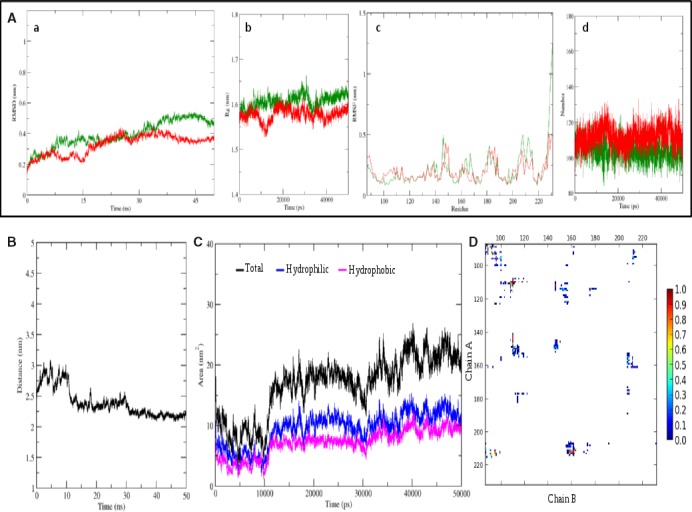
Dimer interface of CagV periplasmic domain. (A) MD Simulation of CagV dimer (Chain A: Green, Chain B: Red). (a) RMSD with reference to starting structure. (b) Radius of gyration. (c) Residue wise RMSF for 50 ns run. (d) Number of intrachain hydrogen bonds. (B) Average distance between COM of chain A and chain B. (C) Buried total, hydrophilic, and hydrophobic solvent accessible surface area at the dimer interface throughout MD simulation. (D) Interchain contact map from 15 ns onwards. Least and most conserved contacts are shown in blue and red.

The CagV dimer was modeled by the structure superimposition of its monomer on two chains of *A. tumefaciens*‐VirB8 (Fig. 7F). In a 50‐ns simulation, we observed a little deviation in RMSD value of individual chains with reference to their starting structure (Fig. 8A). Radius of gyration of individual chain shows some fluctuation in the first 15 ns but converges afterward and starts fluctuating around 1.55–1.6 nm which is very close to the observed Rg value of 1.6 nm, after convergence in the case of monomer simulation (Figs 8A and 7B). Amino acid RMSF value of each individual chain of dimer throughout the simulation is almost similar to the observed RMSF in the last 20‐ns simulation of the monomers (Figs 8A and 7B). After convergence of both, monomer and individual chains of dimer form comparable numbers of intrachain hydrogen bonds (Figs 8A and 7B). These comparative studies suggest that dimerization of CagV seems to introduce very little or no change in the compactness, stability, intrachain hydrogen bond‐forming pattern of each individual units. The plot of distance between the center of mass (COM) of individual chain shows a sharp decrease of around 1 nm between 10 and 20 ns. It is followed by another small decrease around 28–30 ns. For the rest of the period, distance is converged and fluctuate around 2.25 nm (Fig. 8B). This shows that from the reference structure, the individual chain has moved close to each other, getting stabilized at around 2.25 nm after 30 ns. Based on these observations, we confirm that an optimized stable structure of CagV dimer is obtained.

**Figure 9:**
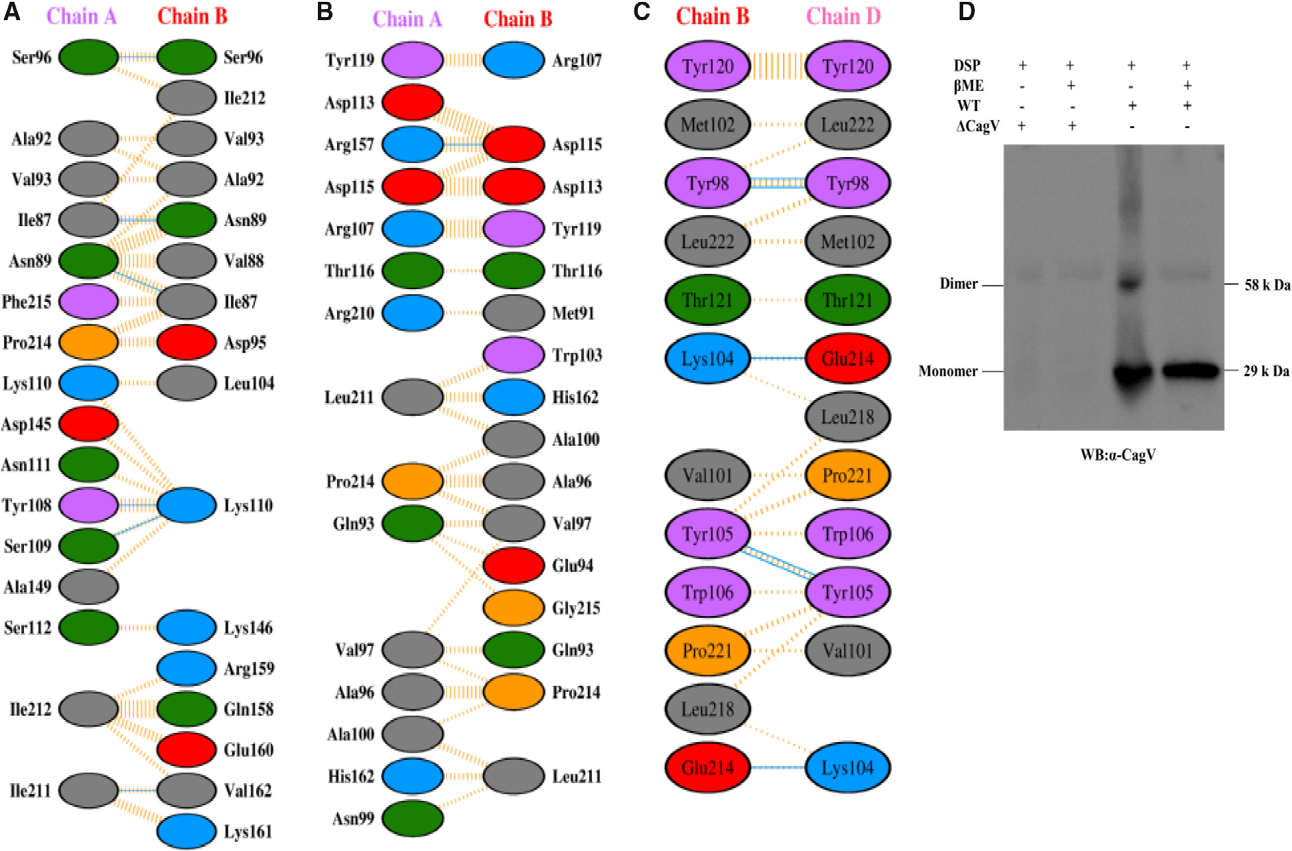
Interactions at the dimer interface of VirB8 homologs and formation of CagV homodimer. (A) DIMPLOT of CagV dimer interface. (B) DIMPLOT of *A. tumefaciens*‐VirB8 (Chain A and B, PDB ID: 2CC3). (C) *B. suis*‐VirB8 (Chain B and D, PDB ID: 4AKZ). Interacting residues of both chains are labeled. Amino acid residues are colored based on side chain property. Green: polar uncharged; cyan: polar positively charged; red: polar negatively charged; yellow: special amino acids; pink: aromatic side chain; hydrophobic side chains. Hydrogen bonds are shown by cyan line. Yellow dashed lines represent hydrophobic contacts. (D) Western blot showing CagV dimer. DSP cross‐linked *H. pylori* cell extracts from wild‐type *H. pylori* and Δ*cagV* strains were separated in SDS/PAGE and western blotted using anti‐CagV antibody generated in mice. Monomer and dimer are indicated on the figure.

A comparative study of the dimer interfaces of *A. tumefaciens*‐VirB8 and *B. suis*‐VirB8 showed that the dimer interface buries over 1700 Å^2^ accessible surface area at their interface 35, 55. We calculated the buried accessible surface area at the interface. The total buried, hydrophilic, and hydrophobic surface area was found to be 2033.313 Å^2^, 1140.546 Å^2^, and 892.471 Å^2^ (Fig. 8C). The total buried surface of the CagV dimer is slightly more than the reported areas for the *A. tumefaciens*‐VirB8 and *B. suis*‐VirB8 proteins. The binding energy between respective chains of the *A. tumefaciens*‐VirB8, *B. suis*‐VirB8, and CagV was found to be −11.212 kcal·mol^−1^, −15.910 kcal·mol^−1^, and −11.180 kcal·mol^−1^, respectively. The contacts between the residues at the dimer interface from 31 ns onward were calculated for each frame and plotted on the basis of frequency to identify conserved contacts (Fig. 8D). At a consensus cutoff of 90%, we found 22 conserved contact pairs between both the chains (Table 3). Contact forming residues Y108 and V144 are strictly and A92 and L104 are moderately conserved (Fig. 6 and Table 4). I211 and I212 are part of the conserved motif 210‐LxxNPxGxxV‐219 which is proposed to position the α‐helix to support dimerization 35. At the dimer interface of *A. tumefaciens*‐VirB8; *B. suis*‐VirB8; and CagV 1, 6, and 6 hydrogen bonds; 25, 19, and 28 hydrophobic contacts; and 78, 80, and 54 non‐bonded interactions were found, respectively (Fig. 9A–C). As essential amino acids of *A. tumefaciens*‐VirB8 V97, P214, and *B. suis*‐VirB8 M102 are involved in forming hydrophobic contacts. *B. suis*‐VirB8 Y105 is involved in hydrogen bonding and hydrophobic contacts with itself (Fig. 9B,C), similarly, aligned residues of CagV to these reported essential residues form similar type of interactions (Figs 6 and 9A). Moderately conserved CagV V93, aligned to *A. tumefaciens*‐VirB8 V97 and *B. suis*‐VirB8 M108 forms hydrophobic contacts. CagV S96, aligned to *B. suis*‐VirB8 Y105 forms hydrogen bond and hydrophobic contacts. CagV P214 forms hydrophobic contacts at the dimer interface (Figs 6 and 9A). Thus, the comparative study suggests that the dimer interface of CagV is almost similar to that of the other two homologs and might form homodimer under a physiological condition. With these notions we performed chemical cross‐linking of wild‐type *H. pylori* extract using homo bifunctional DSP and analyzed the product in SDS/PAGE followed by western blotting using anti‐CagV antibody. As shown in Fig. 9D, CagV forms a dimer like VirB8 homologs.

**Table 3:** Table depicting conserved contacts at the dimer interface of CagV

Chain A	Chain B	Frequency/Consensus rate
S112	K110	1
S109	K110	1
K110	K110	1
D145	K110	1
N89	I87	1
N111	K110	1
K110	S109	0.99
I87	N89	0.99
A149	K110	0.99
Y108	K110	0.98
K110	L104	0.97
K110	N111	0.97
I211	K161	0.97
F143	K110	0.95
Q142	K110	0.95
D145	K146	0.95
V144	K110	0.94
S112	Y108	0.94
S112	K146	0.94
I212	K161	0.90
N89	A92	0.90
A92	A92	0.90

**Table 4:** Conserved residues of CagV. Strictly conserved residues (conserved in all three homologs). Moderately conserved residues (conserved in any two homologs)

Strictly conserved residues	V102, Y108, R141, V144, T175, T195, Yl98, N213, P214, G216, V219
Moderately conserved residues	E91, A92, V93, V94, S99, V102, L104, L113, D118, S127, T130, N139, V147, S115, N156, F172, K177, T179, D181, T182, T190, I196, S106, I212, F217, Q223

### CagV has multiple interacting partners

**Figure 10:**
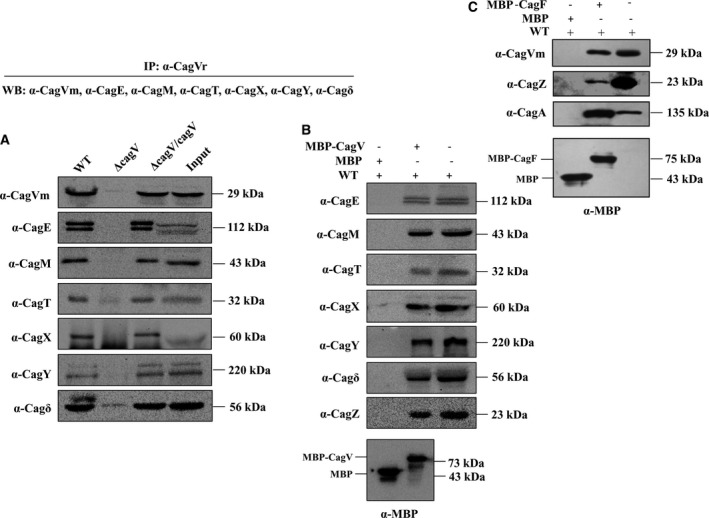
Western blots showing multiple interacting partners of CagV. (A) Co‐immunoprecipitation of CagE, CagM, CagT, CagX, CagY, and Cagδ by anti‐CagV antibody along with CagV from cell extracts of wild‐type *H. pylori* and Δ*cagV/cagV* strains. (B) MBP pull‐down showing interaction of MBP‐CagV with native CagE, CagM, CagT, CagX, CagY, Cagδ, and CagZ from *H. pylori* cell extract. (C) MBP pull‐down showing interaction of recombinant MBP‐CagF with native CagV, CagA, and CagZ from *H. pylori* cell extract. MBP alone was used as a negative control. Antibodies used are marked. Vr and Vm indicate anti‐CagV antibody generated in rabbit and mice, respectively.

It is well documented that VirB8 interacts with a number of VirB proteins and helps in the assembly of the VirB/D4 transporter 28, 51, 57, 58, 59, 60, 61. Since CagV is a predicted homolog of the VirB8, we searched for its interacting partners including CagA by IP using anti‐CagV antibody. IP was performed using detergent‐solubilized wild‐type *H. pylori,* Δ*cag*V, and Δ*cag*V/*cagV* cell extracts by anti‐CagV antibody. The immunoprecipitated samples were separated in SDS/PAGE and western blotted using indicated antibodies. As shown in Fig. 10A, CagE, CagM, CagT, CagX, CagY, and Cagδ were found coimmunoprecipitated along with CagV by anti‐CagV antibody from the wild‐type *H. pylori* and Δ*cag*V/*cagV* extracts but not CagF, CagZ, and CagA. No protein was, however, precipitated where the Δ*cag*V extract was used. These results thus suggest that the interaction between CagV and the outer membrane subcomplex proteins (Cagδ, CagY, CagX, CagT, and CagM) and the inner membrane‐associated energy‐providing component CagE (VirB4 homolog). Since CagF and CagZ are also inner membrane‐associated proteins like CagE and CagV, we hypothesized that these proteins could also interact with each other. The failure to detect precipitation of these proteins using anti‐CagV antibody could be due to the fact that proteins in a large complex such as T4SS interact with each other with different affinity and during antigen antibody interaction in IP, conformation of the complex might change leading to the dissociation of weakly associated proteins. However, other possibilities cannot be ruled out. It is worth mentioning that interaction between recombinant CagV and GST‐CagZ has been reported earlier 62. Therefore, to test the notion, we employed an alternative approach to detect the interaction between these proteins. In this approach, MBP pull‐down experiments were performed using purified, naturally soluble recombinant MBP‐CagV and MBP‐CagF as baits, and detergent‐solubilized *H. pylori* extract as prey, separately (Fig. S3) 47. As shown in Fig. 10B, MBP‐CagV pulled down CagE, CagM, CagT, CagX, CagY, Cagδ, and CagZ from the wild‐type *H. pylori* extract reproducibly, supporting the IP data. Similarly, MBP‐CagF was able to pull‐down CagV efficiently along with CagA and CagZ from the wild‐type *H. pylori* extract (Fig. 10C). It is worth mentioning that the interaction of CagF with CagA has been reported earlier 46, 47. As expected, no protein was pulled down by MBP alone. In order to identify direct interacting partners of CagV, IP was performed using extracts prepared from different Cag mutant strains by anti‐CagV antibody. The precipitated proteins were separated in SDS/PAGE and western blotted using indicated antibodies. As shown in Fig. S4, unlike in the cases of Δ*cagT*, Δ*cagM*, and Δ*cag*δ mutant strains, in the absence of CagX (Δ*cagX*), anti‐CagV antibody failed to coimmunoprecipitate the outer membrane subcomplex proteins suggesting that the interaction between CagV and the outer membrane subcomplex proteins might be through CagX. Previously we have also reported interaction of CagE with the outer membrane‐associated core complex through CagV and CagX following IP using anti‐CagE antibody 14. Taking together these results showed that CagV has multiple interacting partners including CagX‐, CagT‐, and Cag‐T4SS‐specific proteins Cagδ, CagM, CagF, and CagZ.

## Discussion

In this study, we have characterized one essential Cag‐T4SS component, CagV, that shares weak sequence similarity with its counterpart VirB8 of *A. tumefaciens*. However, based on its adaptation of the VirB8 like membrane topology in *E. coli,* it is considered as a homolog 20. To decipher its role in the Cag‐T4SS biogenesis and substrate transport to the bacterial cell surface, we have performed biochemical, genetic, microscopic, and bioinformatics analysis of the protein in the context of multifunctional VirB8. *Agrobacterium tumefaciens*‐VirB8 plays a key role in the VirB/VirD4 T4SS assembly, substrate transfer, and pilus biogenesis 19. VirB8 and its homologs are bitopic inner membrane proteins, form homodimers, and interact with multiple VirB components 56, 57, 58, 59, 60, 61. Here, we have investigated its subcellular localization and shown that like *A. tumefaciens*‐VirB8 CagV is also an inner membrane protein (Fig. 1A–E).

Since our initial attempt to identify native CagV dimer failed, we were prompted to perform a comparative sequence analysis of CagV, along with the two of its homologs *A. tumefaciens*‐VirB8 and *B. suis*‐VirB8 and identified the conserved residues (Fig. 6A). Based on the reported crystal structure data of *A. tumefaciens*‐VirB8 and *B. suis*‐VirB8, a homology model of the periplasmic domain of CagV was generated and found to have similar structure and fold (Fig. 7A). Through comparative MD simulation, we predicted the homodimer interface of the CagV and which was found to be similar to that of the templates. Comparative study of the CagV homodimer interface indicates a high probability of homodimerization of CagV. Based on this information we cross‐linked CagV protein and observed self association (Fig. 9D).

Dimerization of a protein provides large interface for multiple protein interactions and stability 22. *Agrobacterium tumefaciens*‐VirB8 has multiple interacting partners like VirB1, VirB4, VirB5, VirB9 etc. 22, 35, 50, 59, 61. Likewise, CagV interacts with the Cag‐T4SS outer and inner membrane proteins (Fig. 10A–C). However, the interaction between CagV and the outer membrane associated subcomplex proteins is through CagX (Fig. S4). Thus, it is the CagV which bridges the inner membrane with the outer membrane subcomplex. This result reconfirms our earlier report of the interaction of CagE with the outer membrane core complex through CagV 14. It is worth mentioning that the interactions between CagV and Cagδ, CagZ, CagT, CagM, and CagN have been shown by the yeast two hybrid (Y2H) experiments and some of the interactions have been confirmed by pull‐down experiment 17, 62.

*Agrobacterium tumefaciens*‐VirB8 is known to direct the polar localization and stability of several VirB proteins and also stabilizes the secretion system 19, 22, 23. However, cellular localization of CagX and CagT on the bacterial cell surface is found to be independent of CagV (Figs 3A‐D and 4). CagV is also not required for the stability of several essential Cag‐T4SS proteins (Fig. 2A,B). It only partially affects the stability of CagI, a protein involved in the Cag‐T4SS pilus formation (Fig. 2A,B). CagI interacts with CagH and CagL and is required for the Cag‐T4SS pilus formation 29, 63. We have tested interaction of CagV with CagI but they do not interact. These results thus indicate that CagV is not involved directly in stabilizing CagI but might be required to retain the intact Cag‐T4SS structure which in turn affects CagI.

*Agrobacterium tumefaciens*‐VirB8 interacts with the VirD2 and the single‐stranded T‐DNA substrates, which in turn passage the DNA–protein complex from the cytoplasm to the periplasm 59, 64, 65. Substrate transfer to the *A. tumefaciens*‐VirB8 requires the energizing ATPase VirD4, VirB4, and VirB11 together with the outer membrane core components VirB7, VirB9 and VirB10 in the periplasm 58. Surprisingly, however, unlike *A. tumefaciens*‐VirB8, CagV does not interact with the substrate. Nonetheless, Cag‐T4SS substrate CagA interacts with the inner membrane‐associated chaperone CagF and coupling protein Cagβ, respectively 46, 48. Earlier, Jurik *et al*. demonstrated that CagZ interacts with Cagβ 48. CagV on the other hand interacts with CagF, CagE, and CagZ. CagE has also been reported to interact with Cagβ 14. Gene knockout study has shown that CagF is essential for the translocation of CagA into the host cells 5. Hence, it is proposed that CagF interacts with CagA at the cytoplasmic membrane which in turn recruits CagA into the active secretion system at the cytoplasmic face of the bacterium 46, 47, 48. However, how does CagF assist in CagA recruitment into the secretion system and/or what are the other interacting partner(s) of CagF is not known. Recently, Frick‐Cheng *et al*. have shown the interactions between CagF and the outer membrane subcomplex proteins 18. However, they were unable to detect interaction between CagF, CagZ, and CagV. This could be due to the dissociation of weakly interacting partners of CagF during IP reaction. But other possibilities cannot be ruled out. Nonetheless, following Y2H screening, the interaction between CagF and CagY has been reported 17, 62. Busler *et al*. also reported an interaction between recombinant CagV and GST‐CagZ 62. Pinto‐Santini and Salama also demonstrated interactions between Cagδ and a number of Cag‐T4SS components like CagT, Cagβ, CagD, Cagγ, CagA, CagF, and CagY etc. following immuno‐purification of Cag proteins using anti‐Cagδ antibody and subsequent mass spectrometry 66. We have earlier shown the interaction between CagV and the energizing component CagE that is essential for CagA translocation 14. We, therefore, suggest that once Cag‐T4SS is activated, energizing component CagE might modulate CagV at the cytoplasmic membrane, which in turn assists CagF to recruit CagA into the secretion system. This notion is supported by the experimental data that in the absence of CagV, CagA translocation across the bacterial membrane is stalled and CagA cannot reach to the cell surface. However, when *cagV* function is complemented, CagA regains ability to cross the membrane barrier (Figs 5 and S2). Other interpretations also cannot be ruled out, like in the absence of CagV, functional Cag‐T4SS is not formed. Hence, although CagV is considered to be a VirB8 homolog, it lacks a few prominent functional attributes of its counterparts. The present study, therefore, may aid in explaining the structural and functional diversity of Cag‐T4SS from its counterparts.

## Author contributions

GM, MS, NK, and AK conceived and designed the experiments. NK, MS, AK, and RK performed experiments. GM, MS, NK, AK, and NS analyzed the data. GM and RKT contributed reagents. GM, MS, and NK wrote the manuscript.

## Supporting information


**Fig. S1.** Western blots showing specificity of polyclonal anti‐CagV antibody.
**Fig. S2.** IFM showing localization of CagA in wild‐type *H. pylori*, Δ*cagV*, and Δ*cagV/cagV* strains.
**Fig. S3.** SDS/PAGE showing purified MBP‐tagged CagV and CagF.
**Fig. S4.** CagV interacts to the outer membrane subcomplex through CagX.
