# Supplementary material for: Analyzing the role of CagV, a VirB8 homolog of the type IV secretion system of Helicobacter pylori
Source: FEBS Open Bio. 2017 May 24;7(7):915–33. doi: 10.1002/2211-5463.12225 (PMC5494299; doi:10.1002/2211-5463.12225)
Supplement: Supplementary file 1 — Fig. S1. Western blots showing specificity of polyclonal anti‐CagV antibody. Fig. S2. IFM showing localization of CagA in wild‐type H. pylori, ΔcagV, and ΔcagV/cagV strains. Fig. S3. SDS/PAGE showing purified MBP‐tagged CagV and CagF. Fig. S4. CagV interacts to the outer membrane subcomplex through CagX. [file FEB4-7-915-s001.pdf]

Analyzing the role of CagV, a VirB8 homologue of the type IV secretion system of *Helicobacter pylori*

Navin Kumar[1,2#], Mohd Shariq[1,3#], Amarjeet Kumar[4], Rajesh Kumari[1], Naidu Subbarao[4], Rakesh K. Tyagi[1], Gauranga Mukhopadhyay[1*]

1. Special Centre for Molecular Medicine, Jawaharlal Nehru University, New Delhi, India.
2. Present address: School of Biotechnology, Gautam Buddha University, Yamuna Expressway, Greater Noida, Gautam Budh Nagar, Uttar Pradesh, India.
3. Present address: School of life Sciences, Jawaharlal Nehru University, New Delhi, India.
4. School of Computational and Integrative Sciences, Jawaharlal Nehru University, New Delhi, India.

[#] These authors contributed equally to this work

E.mail: [*]gmukho1@gmail.com (GM)

[*]Address for correspondence: Gauranga Mukhopadhyay, Special Centre for Molecular Medicine, Jawaharlal Nehru University, New Delhi, India, 110067. e-mail: gmukho1@gmail.com

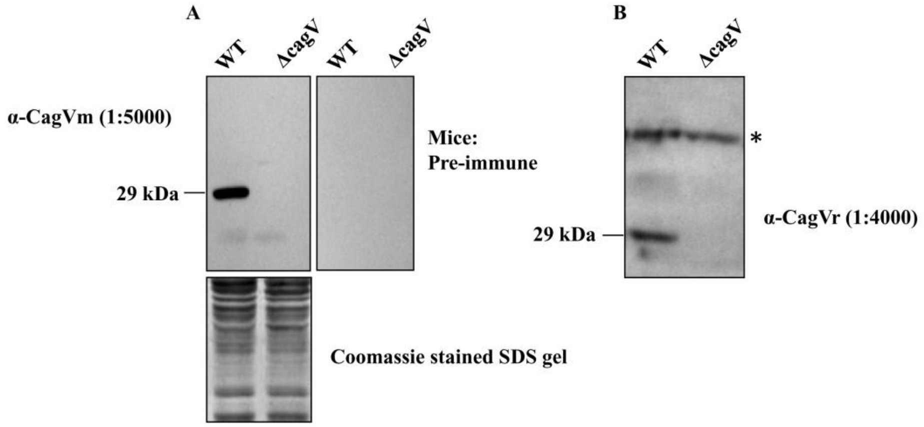

Fig. S1. Western blots showing specificity of polyclonal anti-CagV antibody. Wild-type *H. pylori* and Δ*cagV* cell extracts were resolved in SDS-PAGE and Western blotted by mice anti-CagV antibody (α-CagVm) **(A)** and rabbit anti-CagV antibody (α-CagVr) (**B**), * indicates non-specific protein band detected by anti-CagVr antibody. Mice pre-immune serum was used as a negative control. SDS-PAGE was stained with coommasie dye to show the equal loading of the samples. Titre of individual antibodies is indicated.

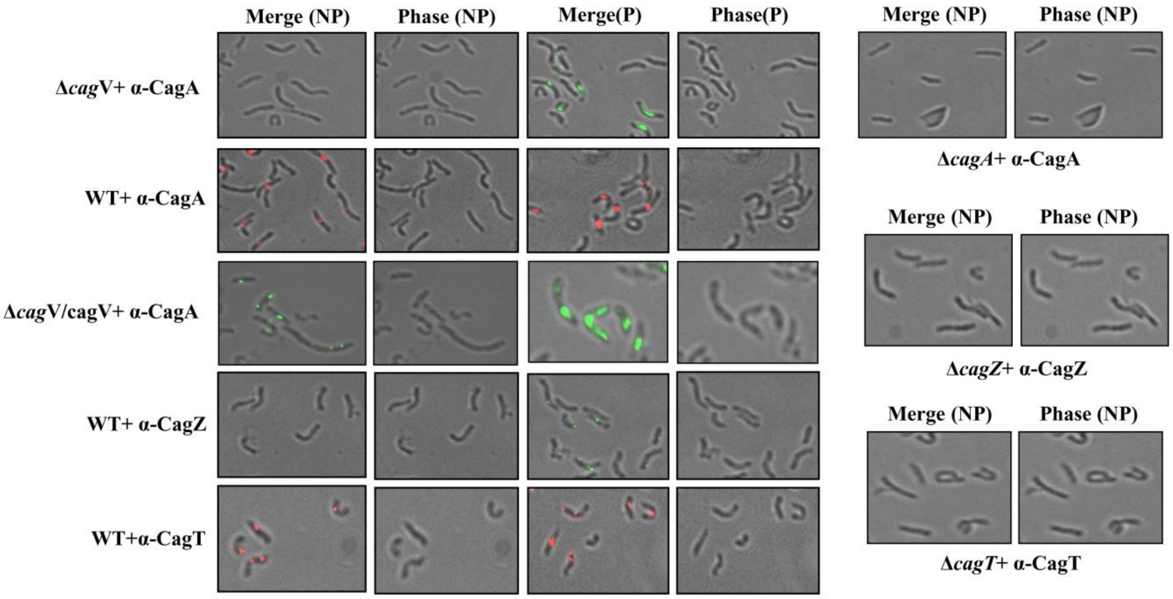

Fig. S2. IFM showing localization of CagA in wild-type *H. pylori*, Δ*cagV* and Δ*cagV/cagV* strains. Δ*cagA*, Δ*cagZ* and Δ*cagT* mutant cells were tested to show specificity of anti-CagA, anti-CagZ and anti-CagT antibodies, respectively. All the bacterial cells were harvested from BHI-agar plate, fixed and one set was permeabilized with 0.2% TritonX-100. NP and P stand for non-permeabilized and permeabilized cells, respectively. Primary antibodies used in IFM are indicated. Alexa fluor 488 (green colour) and Alexa fluor 594 (red colour) conjugated secondary antibodies were used for immune detection. Out of 460 wild-type *H. pylori* cells (from three different experiments) tested, CagA specific fluorescent signals were detected in 290 cells under NP and out of 470, 300 cells showed fluorescence under P conditions. Similarly, out of 640 Δ*cagV* cells tested, CagA specific signals were detected in 11 cells under NP and out of 355, 270 cells showed fluorescence under P conditions. However, in the case of complemented strain Δ*cagV/cagV*, **o**ut of 430 cells (from three different experiments) tested, CagA specific fluorescence signals were detected in 270 cells under NP and out of 410, 300 cells showed fluorescence under P conditions.

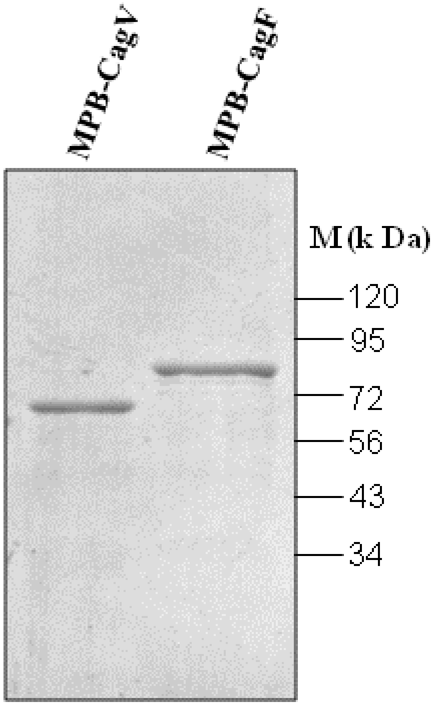

Fig. S3. SDS-PAGE showing purified MBP tagged CagV and CagF. M- Indicates molecular size standards. SDS-PAGE was stained with coomassie brilliant blue.

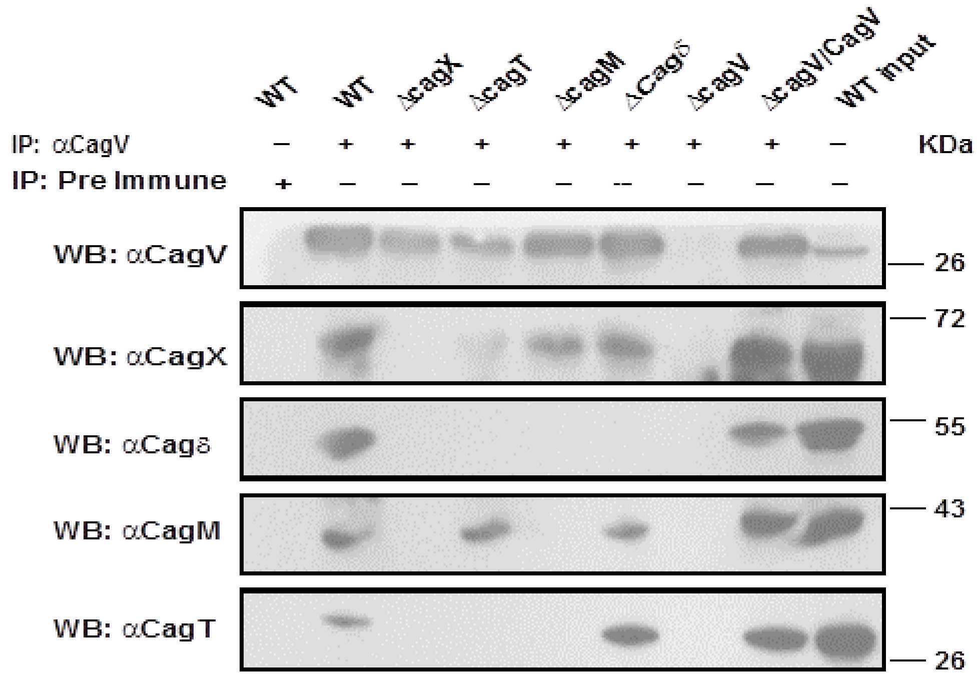

Fig. S4. CagV interacts to the outer membrane sub-complex through CagX. Immunoprecipitations were performed by using anti-CagV antibody and pre-immune serum with different isogenic mutant strains of *cag*-PAI as marked on the figure. Samples were boiled in SDS-sample buffer, separated in SDS-PAGE and Western blotted. Antibodies used are indicated on the figure. Anti-CagVr antibody was used in immunoprecipitation and western blotting. WB: western blotting, IP: immunoprecipitation.
